# Locating Mandibular Foramen in Children with Mandibular Retrognathism in Mixed Dentition

**DOI:** 10.15171/joddd.2015.014

**Published:** 2015-06-10

**Authors:** Mehrsa Paryab, Maryam Ahmadyar

**Affiliations:** ^1^Assistant Professor; Department of Pediatric Dentistry, Faculty of Dentistry, International Campus, Tehran University of Medical Sciences, Tehran, Iran; ^2^General Dental Practitioner, Tehran, Iran

**Keywords:** Foramen, location, mandible, mixed dentition, retrognathism

## Abstract

***Background and aims.*** One of the most common reasons forthe inferior alveolar nerve block anesthesia failure is the variation in mandibular foramen location. The aim of this study was to assess the location of mandibular foramen in children with mandibular retrognathism in comparison to children with normal skeletal occlusion in the mixed dentition.

***Materials and methods.*** One hundred and twenty panoramic radiographs of patients in mixed dentition period, undergoing orthodontic treatment, were selected based on inclusion criteria, skeletal occlusion and stage of dental development. The radiographs were divided into two groups: I: 60 panoramic radiographs of patients with normal skeletal occlusion (15 in each of the Hellman dental age stages); II: 60 panoramic radiographs of patients with mandibular retrognathism (15 in each of the Hellman dental age stages). The radiographs were traced and the linear distance from the mandibular foramen to the borders of the mandibular ramus and its angular position were identified. The measurements were compared between the two groups and among the four dental age groups by t-test, ANOVA and post hoc tests.

***Results.*** No statistically significant differences werefound between the patients with normal skeletal occlusion and patients with mandibular retrognathism (P>0.05). Statistical tests showed significant differences in the vertical location of mandibular foramen and gonial angle between the four dental age groups (P<0.05).

***Conclusion.*** Mandibular retrognathism does not have a significant impact on the location of the mandibular foramen in the mixed dentition period. The child’s dental age would be considered in the localization of the mandibular foramen.

## Introduction

Inferior alveolar nerve block technique is the most commonly used method used in dentistry for pulp treatment and extraction of mandibular primary or permanent teeth.^1,2^ Variations in the position of mandibular foramen (MF) is one of the main reasons associated with an increased failure rate of this thechnique.^3,4^ Olsen suggested that the MF in primary dentition is located below the occlusal plane.^1^ Benham conversely recommended that in the primary dentition, anesthesia solution should be injected at the same level or slightly above the occlusal plane.^5^

As the child grows up, different growth patterns of the mandible and tooth eruption processes lead to variations in the location of the MF.^6^ Different studies have evaluated the location of MF in individuals with different ages and races.^3,7-12^ Location of MF has also been assessed in patients with skeletal malocclusion and significant differences have been shown in the antero-posterior position of MF and distance of MF from the occlusal plane in adult patients with mandibular prognathism.^13,14^ The pattern of mandibular growth can be affected by the morphology of the face.^15,16^This study was designed with the hypothesis that different occlusal and gonial angles in different patterns of mandibular growth can possibly affect the linear distance and location of MF in relation to intraoral landmarks. The aim of this study was to assess the location of MF in children with mandibular retrognathism in comparison to children with normal skeletal occlusion in the mixed dentition.

## Materials and Methods

This case-control study was conducted on the panoramic radiographs of patients who were undergoing orthodontic treatment in the Dental School in Zahedan in 2012. Patients with normal skeletal occlusion and patients with mandibular retrognathism were selected based on their radiographs. The panoramic radiographs were related to pre-orthodontic treatment periods and had all been exposed with the same x-ray machine (CC- Planmeca 2002, Magnification: ×1.2). Radiographs containing gross distortion, non-anatomical radiopacities/radiolucencies, no visible anatomic structures of jaws/infraorbital rims and absence of one of the posterior teeth were excluded from the study. It was confirmed that there were no histories of trauma, surgery and face and neck disorders in the patients.

In addition, patients’ dental ages on the panoramic radiographs were determined based on Hellman dental development stages (Table 1). In each type of skeletal occlusion, fifteen radiographs related to each of the age groups III A, III B, III C and IV A were selected. The groups IIA and IIC were not considered in the sampling of this study because in children aged 3-7, orthodontic treatments for Class II or even Class I malocclusions are usually postponed until the late stages.

**Table 1:** Division of samples into Hellman's dental developmental stages based on dental age and clinical findings

**Group**	**Dental age**	**Hellman’s stages**	**Characteristic**
1	3-4 yrs	IIA	Completion of primary occlusion
2	5-7 yrs	IIC	Eruptive phase of permanent first molar or incisor
3	7-9 yrs	IIIA	Eruption of permanent first molar or incisors completed
4	9-12 yrs	IIIB	Exchange phase of lateral teeth
5	11-12 yrs	IIIC	Eruptive phase of permanent second molar
6	12-13 yrs	IVA	Eruption of permanent second molar completed

Finally, a total of 120 panoramic radiographs were included in the study. The selected subjects were informed and consent was obtained from their parents.

**Figure 1.**
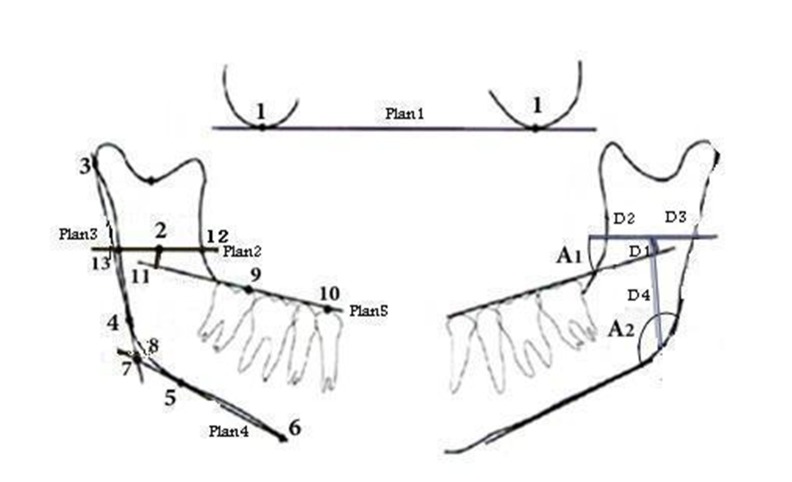
Reference points and planes on the left side of the figure and distances and angles measured on the right side of the figure.

Prior to tracing of the panoramic radiographs, the type of skeletal occlusions was confirmed using McNamara analysis on the lateral cephalometeric radiographs.^17^ Three examiners were prepared for tracing according to the points, planes and angles defined in the Kanno’s study^11^ presented in Figure 1(for clarity, the points and planes have been shown on the left side of the figure and the measured distances and angles have been shown on the right side of the figure). The measurements in the Kanno’s study are suitable for clinical applications. The calibrated examiners that were blinded to the group classifications traced the panoramic radiographs independently. In order to decrease visual error rates, only five radiographs were traced per day. Considering the similarity in MF position between both sides of the mandible,^4,7,9,18^ the right side of the mandibles was selected tracing. Points and planes were traced on matte acetate papers. Linear variables were defined as distances of the MF from the occlusal plane (D_1_), anterior and posterior borders of the ramus and gonial area (D_2_, D_3_ and D_4_, respectively). Angular variables were the angle between MF and the occlusal plane and the gonial angle (A_1_ and A_2_, respectively).

The test-retest repeatability (at a two-week interval) of the measurements was determined for each examiner by kappa coefficient and our results showed a high level of agreement between the test-retest responses and a weighted kappa of 0.93.

### Statistical Analysis

The means of the data collected by the three observers were analyzed using t- test, ANOVA and post hoc tests. Statistical significance was defined at P<0.05.

## Results

One hundred and twenty panoramic radiographs (60 patients with normal skeletal Class I occlusion and 60 patients with mandibular retrognathism) were included in the study. Group I (patients with normal skeletal occlusion) included 34 girls and 26 boys with an age range of 7-14 and a mean age of 9.8±1.76 years. Group II (patients with mandibular retrognathism) included 42 girls and 18 boys with an age range of 7-15 and a mean age of 9.48±1.50 years.

K_2_ and t-test analyses showed that the two groups were similar in terms of gender and age distributions (P=0.368).

The mean of linear and angular measurements of the two groups were compared by t-test analysis. No statistically significant differences were found (P>0.05) (Table 2).

**Table 2:** The results of comparison of the means of linear and angular measurements between groups I and II

**Measurements**	**Group I**	**Group II**	**P-value**
**A_1_**	16.24 (‏±6.02)	15.80 (‏±6.26)	0.692
**A_2_**	127.42 (‏±5.32)	128.06 (‏±5.11)	0.506
**D_1_**	3.69 (‏±2.11)	4.01 (‏±2.14)	0.409
**D_2_**	20.34 (‏±2.70)	20.64 (‏±2.19)	0.504
**D_3_**	12.92 (‏±1.76)	13.05 (‏±2.14)	0.706
**D_4_**	27.19 (‏±3.06)	27.56 (‏±3.48)	0.538
**Group I:** Patients with normal skeletal occlusion; **Group II:** patients with mandibular retrognathism; **A_1_:** Occlusal plane angle; **A_2_:** Gonial angle; **D_1_:** Distance of MF from the occlusal plane; **D_2_:** Distance of MF from the anterior border of the ramus; **D_3_:** Distance of MF from the posterior border of the ramus; **D_4_:** Distance of MF from the gonial area.			

The measurements were also compared among the four dental age groups and in girls and boys, separately. ANOVA showed significant differences in D_1_ (distance of MF from the occlusal plane), D_4_ (distance of MF from the gonial area) and A_2_ (gonial angle) measurements between the four age groups (P<0.05) (Table 3). Post hoc statistical tests showed that in the measurements mentioned above, stage IVA showed a significant difference compared to the other stages (Table 4). As presented in Table 4, the distance of MF from the occlusal plane and gonial angle showed a significant decrease around stage IVA in comparison to the preceding stages. On the contrary, a significant increase was found in the MF distance from the gonial area in this stage.

**Table 3:** The results of ANOVA in comparison of the means of linear and angular measurements between four dental age stages

**Measurements**	**IIIA**	**IIIB**	**III C**	**IVA**	**P-value**
**A_1_**	17.17 (‏±6.35)	14.52 (‏±5.84)	17.02 (‏±5.77)	15.38 (‏±6.35)	0.264
**A_2_**	129.37 (‏±4.89)	127.97 (‏±4.54)	128.59 (‏±5.32)	125.16 (‏±5.27)	0.009
**D_1_**	4.68 (‏±2.14)	3.85 (‏±2.27)	4.08 (‏±1.92)	2.80 (‏±1.75)	0.005
**D_2_**	20.11 (‏±2.35)	20.14 (‏±2.28)	20.37 (‏±2.52)	21.31 (‏±2.57)	0.184
**D_3_**	12.99 (‏±1.49)	13.03 (‏±2.00)	12.36 (±2.08)	13.53 (‏±2.07)	0.136
**D_4_**	26.72 (‏±2.60)	26.22 (‏±2.44)	26.44 (‏±2.89)	30.02 (‏±3.50)	0.00
**A1:** Occlusal plane angle; **A2:** Gonial angle; **D1:** Distance of MF from the occlusal plane; **D2:** Distance of MF from the anterior border of the ramus; **D3:** Distance of MF from the posterior border of the ramus; **D4:** Distance of MF from the gonial area.					

**Table 4:** The results of post hoc tests in comparison of the means of linear and angular measurements be-tween four dental age stages

**Measurements**	**IIIA**	**IIIB**	**III C**	**IVA**
**A_1_**				
IIIA	—	0.336	1.000	0.661
IIIB	0.336	—	0.391	0.946
IIIC	1.000	0.391	—	0.722
IVA	0.661	0.946	0.722	—
**A_2_**				
IIIA	—	0.707	0.935	0.008
IIIB	0.707	—	0.964	0.134
IIIC	0.935	0.964	—	0.045
IVA	0.008	0.134	0.045	—
**D_1_**				
IIIA	—	0.389	0.663	0.003
IIIB	0.389	—	0.971	0.192
IIIC	0.663	0.971	—	0.073
IVA	0.003	0.192	0.073	—
**D_2_**				
IIIA	—	1.000	0.974	0.224
IIIB	1.000	—	0.983	0.246
IIIC	0.974	0.983	—	0.443
IVA	0.224	0.246	0.443	—
**D_3_**				
IIIA	—	1.000	0.583	0.698
IIIB	1.000	—	0.534	0.744
IIIC	0.583	0.534	—	0.089
IVA	0.698	0.744	0.089	—
**D_4_**				
IIIA	—	0.909	0.982	0.000
IIIB	0.909	—	0.991	0.000
IIIC	0.982	0.991	—	0.000
IVA	0.000	0.000	0.000	—
**A1:** Occlusal plane angle; **A2:** Gonial angle; **D1:** Distance of MF from the occlusal plane; **D2:** Distance of MF from the anterior border of the ramus; **D3:** Distance of MF from the posterior border of the ramus; **D4:** Distance of MF from the gonial area.				

## Discussion

In inferior alveolar nerve block anaesthesia, it is essential that the tip of the needle be placed near the mandibular foramen. Different results have been obtained regarding the anatomical location of mandibular foramen in relation to intraoral landmarks.^3,4^ This subject has also been investigated in the Iranian population.^7,8^It appears that different remodelling patterns of the ramus and other parts of the mandible may affect the final location of the mandibular foramen. Modelling of various parts of the mandible occurs along with the tooth eruption and shedding.^9^ This study was designed with the hypothesis that various occlusal and gonial angles in different growth patterns in children with mandibular retrognathism can affect the linear distance of MF from intraoral landmarks and should be considered in the injection of local anesthesia solution. A small group of Iranian children were included in this study.

In this study, panoramic radiographs from the archived files of patients in Department of Orthodontics were used. Panoramic radiography is one of the convenient techniques to determine the position of MF. In this regard, it has been reported that panoramic radiographs are as reliable as oblique cephalometeric radiographs.^3,12^The location of MF on the panoramic radiographs was determined based on the mandibular canal and triangular opacity of the lingula.^19^ In younger children, the lingula is close to the MF and can be used as a reliable radiographic landmark to determine the location of MF.

The results of the present study showed that the MF is located 3-4 mm above the occlusal plane. Some studies have reported^11^ higher and some others have shown^3,7-9^ lower distances between MF and the occlusal plane. These differences might be attributed to ethnic and racial variations of jaw growth patterns, sampling of the children based on dental age instead of chronological age and also tracing based on different landmarks.

After statistical analysis, no significant differences were observed between the two groups in terms of vertical and horizontal positions of the MF. This means that skeletal retrognathism of mandible during mixed dentition has no significant impact on the position of the MF in relation to ramus borders or the occlusal plane. Lee et al^13^ and Seo et al^14^ showed significant differences in the position of MF between patients with skeletal Class III and patients with a normal skeletal relationship. Their studies were conducted on adult patients; therefore, it is likely that in adulthood, particularly in severe cases of Class II malocclusion, significant differences can be found in the position of MF, and further studies are required in this area.

In comparison of vertical linear and angular measurements between different dental age groups, vertical distance from the MF to the gonial area (D_4_) displayed significant and constant changes along with increasing dental age up to stage IVA. MF distance from the occlusal plane (D_1_) and also gonial angle (A_2_) eventually showed a significant decrease in stage IVA.

There were insignificant changes in the occlusal plane angle (A_1_) from stage IIIA to IVA indicating both increased and decreased values, which might be attributed to constant remodelling of bone due to the active phase of eruption and shedding. The distance of MF from the occlusal plane did not display any significant changes prior to stage IVA. Poonacha and Afsar^3,9^ also reported little changes in this distance and Kanno^11^ found this distance to remain stable only in girls. On the contrary, in some other studies, an increase has been found as the child grows.^7,12^

During the eruption of the lateral incisor teeth (8‒9 years of age), the development of the naso-maxillary complex leads to vertical growth of the mandibular ramus and downward rotation of the mandible. Thereafter, along with the anterior development of the mandibular body, the anterior open bite and rotation of the mandible will be compensated. These changes result in a decrease in the gonial angle. The decrease in the gonial angle and growth of the alveolar process in the canine, premolars and second permanent molar areas cause the upward movement of the occlusal plane. This may be the causative factor resulting in a decrease of the MF distance from the occlusal plane around age 14 (stage IV A). However, at this stage, deposition of bone will occur in the gonial area of the mandible. Therefore, the distance between the MF and the gonial area increases (D_4_). This indicates that the vertical position of the MF is relatively constant from the occlusal plane in spite of the significant increase in the vertical dimensions of the mandible. These changes have been shown in studies on the mandibular growth.^13,14^ Such results are also consistent with those of a study by Poonacha;^9^ such a consistency in the results might be attributed to the similarity in methodologies.

The comparison of horizontal linear and angular variables between different dental age groups showed that the distances of MF from the anterior and posterior borders of the ramus do not change significantly. On the other hand, the horizontal measurements (D_2_, D_3_) did not change in any dental age group of the mixed dentition. Stability in horizontal dimensions has been showed in many studies.^7,9-12^ It has also been found that in the mixed dentition, the distance of MF from the anterior border of the ramus is constantly more than the distance of MF from the posterior border.

The present study was conducted on a small group of children in the mixed dentition stage. The different growth pattern of patients with mandibular retrognathism may continue into the next growth stages after the mixed dentition stage. Therefore, it is suggested that similar studies be designed and performed in adult patients with severe Class II malocclusion.

In addition, the distribution and the number of boys and girls in each of the dental age groups were not similar and sufficient for statistical analysis. A larger sample size is essential in order to distribute equal and efficient numbers of girls and boys in each of the age groups.

## Conclusions

1. Mandibular retrognathism in the mixed dentition stage does not have a significant impact on the location of the mandibular foramen in relation to the clinical landmarks.
2. The child’s dental age would be considered the main factor affecting the location of the mandibular foramen.
